# Unique Responses of Stem Cell-Derived Vascular Endothelial and Mesenchymal Cells to High Levels of Glucose

**DOI:** 10.1371/journal.pone.0038752

**Published:** 2012-06-06

**Authors:** Emily Keats, Zia A. Khan

**Affiliations:** 1 Department of Pathology, University of Western Ontario, London, Ontario, Canada; 2 Metabolism and Diabetes Program, Lawson Health Research Institute, London, Ontario, Canada; University of Bristol, United Kindom

## Abstract

Diabetes leads to complications in selected organ systems, and vascular endothelial cell (EC) dysfunction and loss is the key initiating and perpetuating step in the development of these complications. Experimental and clinical studies have shown that hyperglycemia leads to EC dysfunction in diabetes. Vascular stem cells that give rise to endothelial progenitor cells (EPCs) and mesenchymal progenitor cells (MPCs) represent an attractive target for cell therapy for diabetic patients. Whether these vascular stem/progenitor cells succumb to the adverse effects of high glucose remains unknown. We sought to determine whether adult vascular stem/progenitor cells display cellular activation and dysfunction upon exposure to high levels of glucose as seen in diabetic complications. Mononuclear cell fraction was prepared from adult blood and bone marrow. EPCs and MPCs were derived, characterized, and exposed to either normal glucose (5 mmol/L) or high glucose levels (25 mmol/L). We then assayed for cell activity and molecular changes following both acute and chronic exposure to high glucose. Our results show that high levels of glucose do not alter the derivation of either EPCs or MPCs. The adult blood-derived EPCs were also resistant to the effects of glucose in terms of growth. Acute exposure to high glucose levels increased caspase-3 activity in EPCs (1.4x increase) and mature ECs (2.3x increase). Interestingly, MPCs showed a transient reduction in growth upon glucose challenge. Our results also show that glucose skews the differentiation of MPCs towards the adipocyte lineage while suppressing other mesenchymal lineages. In summary, our studies show that EPCs are resistant to the effects of high levels of glucose, even following chronic exposure. The findings further show that hyperglycemia may have detrimental effects on the MPCs, causing reduced growth and altering the differentiation potential.

## Introduction

Vascular dysfunction is the underlying cause of each of the clinical manifestations of long-term diabetes [1], [2]. It presents as both micro- (cardiomyopathy, retinopathy, nephropathy, neuropathy) and macro-(atherosclerosis) angiopathies [3]. It is now widely accepted that in each target organ, problems arise from sustained hyperglycemia acting directly on the endothelial cells (ECs) [4], [5]. With chronic insult, biochemical alterations occur in the ECs that result in structural and functional variations in blood vessels [1]. Over time, and with the aberration of entire vascular networks, blood flow is altered and tissues become ischemic [6]. This induces a variety of complications that differ depending on the target organ vascular bed. We hypothesized that a balance exists between uncontrolled neovascularization and fibrosis [2], and will shift to one side depending on the tissue microenvironment (i.e. growth factors, matrix proteins) and the intrinsic properties of the ECs, as well as the presence of other risk factors (i.e. hyperlipidemia, hyperinsulinemia).

An intact vascular system is essential for the continued delivery of oxygen and nutrients to the tissue and the removal of waste products- both of which are required to maintain proper tissue functioning. ECs and perivascular cells work together, and are necessary, for the formation of stable and functional vascular networks. With the discovery of postnatal endothelial progenitor cells (EPCs) [7], [8], a new concept of neovascularization has emerged. It is now understood that complete vasculogenesis (i.e. the differentiation of progenitor cells into vascular cells) is able to take place postnatally [9], [10]. With local damage or ischemia, progenitor cells are stimulated to mobilize from the bone marrow and can congregate in areas of injury through the use of cytokines and other homing mechanisms [11]. Once situated, they can incorporate and differentiate into vascular cells in order to restore homeostasis. From this viewpoint, an insufficient number of progenitor cells may cause or contribute to any ischemic disease. The insufficiency in progenitor cells may be caused by a number of factors such as impaired bone marrow release, loss of migratory ability, loss of differentiation potential, or shortened survival time in the peripheral circulation.

The idea of a specific vascular stem cell (VSC) population is one that is steadily gaining recognition. VSCs are a sub-population of CD133+ cells that are able to differentiate into mature cells of the vascular wall [12], [13]. VSCs are predominantly housed in the bone marrow, but can also be derived from the mononuclear cell layer of peripheral blood, making them an easily attainable source of cells. The existence of a common vascular stem/progenitor cell that can be derived from adult human blood samples highlights the feasibility of therapeutic vasculogenesis for long-term diabetic patients. Although we know VSCs may provide the basis for vasculogenesis in a nude mouse model [14], whether they are able to restore vascular homeostasis in an *in vivo* diabetic setting remains to be determined. The effects of high glucose on the functionality of both EPCs and MPCs must be elucidated firstly. To date, the role of MPCs in diabetic complications has not been investigated, and much of the work done on EPCs (short-term colonies; 2–4 days in culture) is confounded by the presence of hematopoietic/monocytic cells within the studies [15], [16]. These ‘early’ cells are characterized by Ulex europaeus agglutinin binding and DiI-labeled acetylated-low density lipoprotein (LDL) uptake [15], [17], [18]. Both of these assays are not specific to ECs [19], [20]. Therefore, a combination of both phenotypic and functional properties must be used to unambiguously identify both EPCs and MPCs so as not to impair the results of the studies. We herein determine the precise role of vascular EPCs and MPCs in a high glucose setting in order to highlight their potential application in diabetes therapy.

## Methods

### Isolation, culture, and differentiation of VSCs

All experiments were approved by the Research Ethics Board at the University of Western Ontario, London Ontario. Normal adult peripheral blood (US Biological; age 26.16±6.79 yrs) and fresh bone marrow samples (Lonza Inc.) were obtained and mononuclear cell fraction was prepared as shown by us previously [21]. To obtain adult blood EPCs (abEPCs), cell suspensions were cultured on FN-coated (FN; 1 µg/cm^2^; Millipore) plates in modified endothelial cell media: complete Endothelial Basal Media-2 (EMB2; Lonza Inc.) supplemented with 35% fetal bovine serum (FBS; Lonza Inc.) and EBM2 SingleQuots (Lonza Inc.). The media was changed every day until colonies began to appear. Thereafter the media was changed every other day. All subcultures were then performed with EBM2/20% FBS/SingleQuots media. Bone marrow samples were cultured on FN-coated plates in DMEM (Invitrogen) media, supplemented with 20% FBS, 1X PSF (antibiotic-antimycotic solution; CellGro Mediatech Inc.), and no additional growth factors. To assess the effect of high levels of glucose on abEPC and bone marrow MPC (bmMPC) differentiation, the media was supplemented with 25 mmol/L glucose from the initial plating. All other experiments were conducted on passage 2–6 cells with 3 technical and 3–5 biological replicates.

Normal human dermal microvascular endothelial cells (HDMECs; CC-2516, Lonza Inc.) and human cord blood-derived EPCs (cbEPCs; derived from cord blood using the same protocol as mentioned above for adult blood; 2C-150A; Lonza Inc.) were used as control for the abEPCs. Human umbilical artery smooth muscle cells (uaSMC) were used as control for the bmMPCs.

### Cell staining

Cultured cells were trypsinized and plated (10,000 cells/cm^2^) on FN-coated 8-chambered slides one day prior to staining to allow for attachment of cells. Immunofluorescence staining for endothelial cell markers was carried out using goat anti-human CD31 (1∶200; Santa Cruz Biotechnology Inc.), rabbit anti-human Von Willebrand Factor (vWF, 1∶200; DakoCytomation), and goat anti-human vascular endothelial (VE)-Cadherin (1∶200; Santa Cruz Biotechnology Inc). For the mesenchymal cells, we used mouse anti-human α-smooth muscle actin (1∶200; Sigma), mouse anti-human CD90 (1∶200; Abcam), mouse anti-human NG2 (1∶200; Abcam), and mouse anti-human platelet-derived growth factor receptor-β (PDGF-Rβ, 1∶200; R&D Systems) antibodies followed by FITC- or Alexa488-conjugated secondary antibodies. Slides were subjected to a nuclei counterstain using DAPI and mounted using Fluoromount mounting medium. Images were taken using Olympus BX-51 fluorescent microscope and Spot Basic software.

### RNA isolation and qRT-PCR

Using RNeasy Mini Plus or Micro Plus (Qiagen, Mississauga, Ontario), total RNA was extracted from the cells grown in culture. Purity of the RNA samples was determined by measuring the absorbance at 260∶280 nm in GeneQuant Spectrophotometer (Pharmacia Biotech). The quantity was determined by Qubit® Broad Range RNA assay in the Qubit® Fluorometer (Invitrogen). cDNA synthesis was performed with 200 ng of RNA using iScript cDNA Synthesis Kit (Bio-Rad Laboratories, Hercules, CA). Primers used for RT-PCR are listed in Table 1. RT-PCR reactions consisted of 10 µL 2X SYBR Advantage qPCR premix (Clontech Laboratories, Inc., Mountain View, CA), 2 µL of both forward and reverse primers (at a 10 µM concentration), 2 µL cDNA, and 6 µL of H_2_O. All reactions were performed for 40 cycles using the following temperature profiles: 95°C for 5 minutes (initial denaturation); 55°C for 10 seconds (annealing); and 72°C for 12 seconds (extension). 18S rRNA was used as the housekeeping gene. PCR specificity was determined by both the melting curve analysis and gel electrophoresis and the data was analyzed by standard curve method.

**Table 1:** Primer sequence information for qRT-PCR.

Gene	Length (bp)	Source (catalogue number)
**Adipogenesis markers**		
CCAAT/enhancer binding protein alpha (C/EBPα)	88 bp	Qiagen (QT00203357)
Peroxisome proliferator-activated receptor γ2 (PPARγ2)	134 bp	5′→3′ ATTGACCCAGAAAGCGATTCC CAAAGGAGTGGGAGTGGTCT
**Chondrogenesis markers**		
NK3 homeobox 2 (Nkx3.2)	100 bp	Qiagen (QT01079582)
Runt-related transcription factor 2 (Runx2)	102 bp	Qiagen (QT00020517)
Sex determining region Y-box 9 (Sox9)	111 bp	Qiagen (QT00001498)
**Endothelial cell markers**		
CD31	144 bp	Qiagen (QT00081172)
CD34	106 bp	Qiagen (QT00056497)
Vascular endothelial growth factor receptor 2 (VEGFR-2)	78 bp	Qiagen (QT00069818)
Vascular endothelial cadherin (VE-cadherin)	109 bp	Qiagen (QT00013244)
Von Willebrand factor (vWF)	108 bp	Qiagen (QT00051975)
**Endothelial cell activation markers**		
Endothelin 1 (ET-1)	166 bp	Qiagen (QT00088235)
Endothelial selectin (E-selectin)	96 bp	Qiagen (QT00015358)
Intercellular adhesion molecule 1 (ICAM1)	84 bp	Qiagen (QT00074900)
**Extracellular matrix** (**ECM**) **proteins**		
Fibronectin	119 bp	Qiagen (QT00038024)
Collagen 1	118 bp	Qiagen (QT00037793)
Collagen 3	95 bp	Qiagen (QT00058233)
Collagen 4	119 bp	Qiagen (QT00005250)
**Mesenchymal cell markers**		
Alpha smooth muscle actin (α-SMA)	83 bp	Qiagen (QT00088102)
Calponin	78 bp	Qiagen (QT00067718)
Myosin heavy chain (MHC)	130 bp	Qiagen (QT00069391)
NG2	128 bp	Qiagen (QT00079884)
Platelet-derived growth factor receptor β (PDGFR-β)	102 bp	Qiagen (QT00082327)
**Osteogenesis markers**		
Osterix (SP7)	120 bp	Qiagen (QT00213514)
Runt-related transcription factor 2 (Runx2)	102 bp	Qiagen (QT00020517)
**Oxidative stress markers**		
Catalase (Cat)	60 bp	Qiagen (QT00079674)
Glutathione peroxidase 1 (GPx)	105 bp	Qiagen (QT00203392)
Heme oxygenase 1 (HO-1)	99 bp	Qiagen (QT00092645)
NADPH oxidase (p22 Phox)	106 bp	Qiagen (QT00082481)
Inducible NOS (NOS2)	92 bp	Qiagen (QT00068740)
Superoxide dismutase 1 (SOD-1)	150 bp	Qiagen (QT01671551)
**Housekeeping gene**		
18S rRNA	149 bp	Qiagen (QT00199367)

### Cell growth assay

The growth of the abEPCs and bmMPCs was assayed by plating the cells in triplicates on FN-coated multiwell plates (Corning Life Sciences) at a density of 2500 cells/cm^2^. The EPCs were cultured in complete EBM2/20% FBS media with or without the addition of high glucose (25 mmol/L; 450 mg/dL). Cell number was determined on days 1, 6, and 12 using Scepter 2.0 Automated Cell Counter (Millipore). The histogram gating was adjusted to specifically measure live cells. bmMPCs were also plated at a density of 2500 cells/cm^2^ in multiwall plates. However, the cells were cultured in DMEM/10% FBS media with or without the addition of 25 mmol/L glucose for 1, 4, and 12 days. The data was presented as cell counts. The results were also confirmed by colorimetric assay utilizing tetrazolium salt reagent (WST-1, Clontech, Mountain View, CA). Following incubation, the absorbance was measured at 450 nm using Multiskan FC Microplate Photometer (Thermo Scientific, USA) with 690 nm absorbance as the reference point.

### Caspase-3 activity

We also tested the effect of high glucose levels on caspase-3 activity (BF3100; R&D Systems) in serum-reduced media (EBM2/1% FBS for endothelial cell types and DMEM/1% FBS for mesenchymal cell types). The cells were cultured for up to 4 days (longer time periods were not possible as serum reduction itself causes cell death) with or without the addition of 25 mmol/L glucose and cell lysates were prepared. To measure caspase-3 activity, a caspase-specific peptide conjugated to p-nitroaniline was added to the lysates and the cleavage product was quantified by measuring absorbance at 405 nm.

### Cell migration assay

A migration assay was performed on both abEPCs and bmMPCs using FN-coated 6.5-mm Transwell inserts with 8.0-μm pores (BD Falcon Cell Culture inserts; BD Biosciences). abEPCs were trypsinized and re-suspended in control (EBM2/1% FBS) or high glucose (EBM2/1% FBS +25 mmol/L glucose) media. One hundred µL of cell suspension was added to the inserts in triplicates, and at a density of 10,000 cells/insert. The lower chambers contained 10 ng/mL basic fibroblast growth factor (bFGF; 233-FB-025, R&D Systems). The cell density used in this assay was optimized for endothelial cells in our pilot studies to provide a robust measure of cell migration. Twenty four hours later, cells on the upper insert were removed and cells in the lower chamber that had migrated through the pore were trypsinized. The cell suspension was centrifuged, re-suspended in media, and added to 96-well plates in order to be measured by the Multiskan FC Microplate Photometer. bmMPC migration was measured similarly but with pre-optimized 25,000 cells/cm^2^. The lower chambers in bmMPC migration experiments contained 10% serum as the chemoattractant.

### MPC differentiation assay

A differentiation assay was conducted on bmMPCs to assess whether they retain their multipotential nature under high glucose conditions. Cells were treated with control or high glucose media (DMEM/10%) for 7 days before the differentiation experiments. To induce differentiation, bmMPCs were seeded at a density of 40,000 cells/cm2 on 12-well plates in specific differentiation media (StemPro® Adipogenesis/Chondrogenesis/Osteogenesis Differentiation media; Invitrogen). Media was changed every other day. RNA was isolated from cells after 7 and 14 days to perform qRT-PCR in order to quantify the differentiation potential. We measured peroxisome proliferator activated receptor-γ2 and C/EBP α for adipogenesis, Runx2 and osterix (SP7) for osteogenesis, and Sox9, Nkx3.2, and Runx2 for chondrogenesis. These transcription factors are essential for the differentiation of multipotential cells into the lineages indicated [22], [23].

### Statistical analysis

The data were expressed as means ± SEM. Where appropriate, analysis of variance (ANOVA) followed by two-tailed student's unpaired t-tests were performed. P values <0.05 were considered statistically significant.

## Results

### Isolation and characterization of abEPCs and bmMPCs

**Figure 1:**
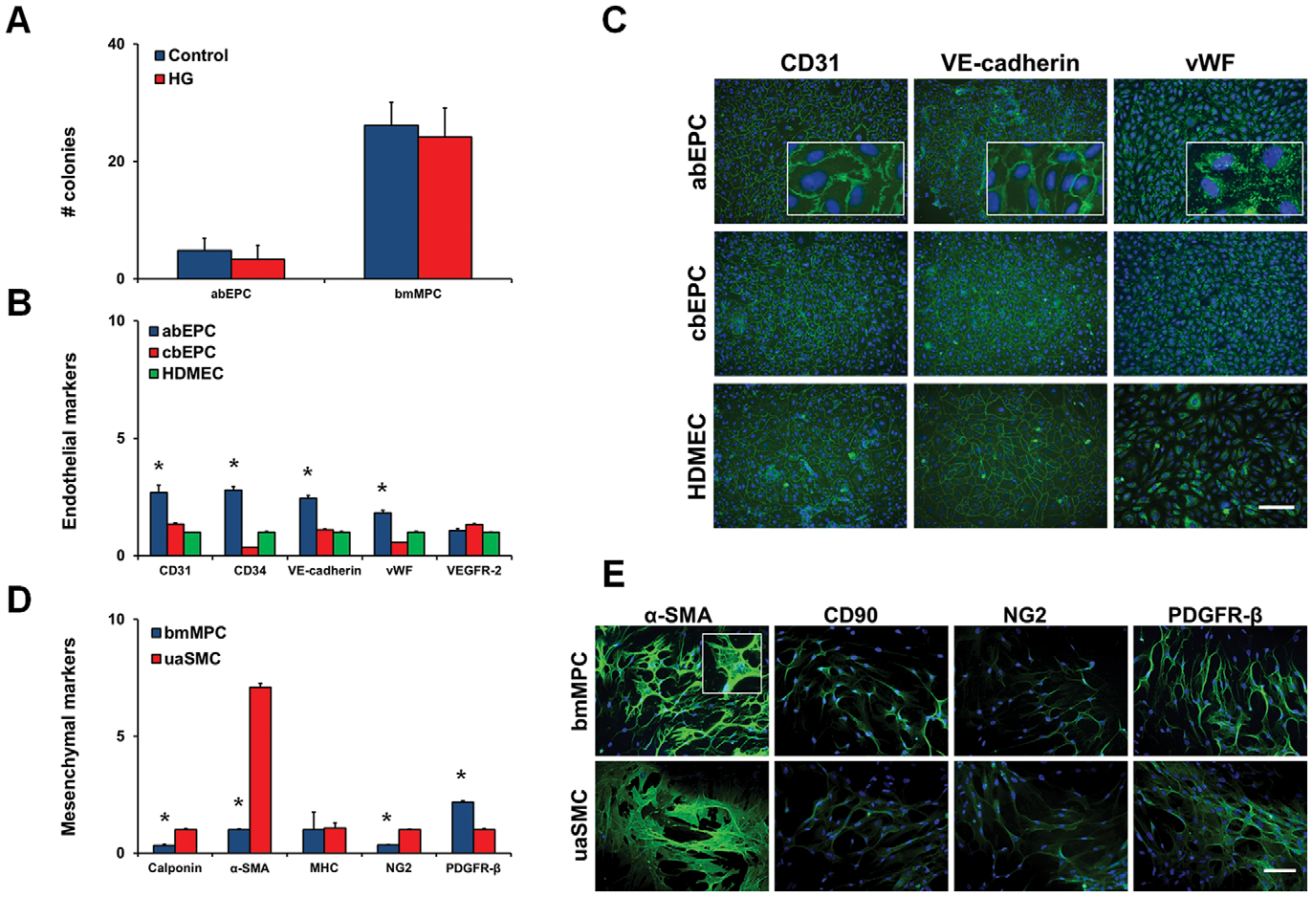
Characterization of human adult blood-derived EPCs (abEPCs) and bone marrow-derived MPCs (bmMPCs). (A) abEPCs (n = 9) and bmMPCs (n = 6) were derived from the mononuclear cell layer of adult peripheral blood samples and bone marrow samples, respectively. No significant change in the number of abEPC and bmMPC colonies was seen with the addition of high glucose (25 mmol/L) to the culture medium. (B) abEPCs were characterized through quantitative RT-PCR analysis for expression of known EC-markers: CD31, CD34, VE-cadherin, vWF, and VEGFR-2 [mRNA data normalized to 18S rRNA and presented relative to HDMECs; *p<0.05 compared to HDMECs; n = 3]. (C) abEPCs were further characterized through immunostaining for antibodies against cell surface markers CD31 and VE-cadherin, and intracellular marker vWF [Blue: DAPI for nuclear staining; Green: Alexa Fluorochrome 488; scale bar represents 100 µm]. (D) Similarly, qRT-PCR analysis of bmMPCs showed expression of mesenchymal markers: calponin, α-SMA, MHC, NG2, and PDGFR-β [data normalized to 18S rRNA and presented relative to uaSMCs; *p<0.05 compared to uaSMCs; n = 3]. (E) MPCs were immunostained for antibodies against membrane-bound proteins CD90, NG2, and PDGFR-β, and cytoplasmic protein α-SMA. [Blue: DAPI for nuclear staining; Green: Alexa Fluorochrome 488; scale bar represents 100 µm].

Culture of the adult blood cells in EBM-2 media supplemented with SingleQuots induced differentiation of the cells into the endothelial lineage (abEPCs). Culture of blood-derived cells in high glucose media did not significantly alter the number of colonies (Figure 1A). No colonies appeared in either control or high glucose level conditions prior to day 14 (data not shown; plates were screened daily using phase contrast microscopy). abEPCs were then characterized through RT-PCR to confirm expression of endothelial cell-selective genes and through immunocytochemistry to properly localize the cellular markers. cbEPCs and HDMECs were used as controls. RT-PCR confirmed the expression of 5 genes of known significance to endothelial cells: CD31, CD34, VEGFR-2, VE-cadherin, and vWF (Figure 1B). The expression of all endothelial-specific genes tested, except for VEGFR-2, was significantly higher in abEPCs as compared to mature HDMECs (Figure 1B). Immunostaining showed both CD31 and VE-cadherin localized to the cell membrane of abEPCs, as anticipated (Figure 1C). vWF, an intracellular protein stored in Weible Palade bodies, showed intracellular localization.

bmMPC colonies appeared within the first week of culture. Similar to the abEPC preparations, high levels of glucose did not significantly affect the number of colonies derived (Figure 1A). MPCs were then subcultured and compared to umbilical artery smooth muscle cells (uaSMCs) for expression of mesenchymal markers. bmMPCs expressed mRNA for all mesenchymal markers tested (Figure 1D). Interestingly, PDGFR-β was highly expressed in bmMPCs as compared to mature uaSMCs (Figure 1D; p<0.05). Other markers of mesenchymal lineage showed significantly lower expression in the bmMPCs. Positive staining was observed for CD90, NG2, PDGFR-β, and α-SMA in bmMPCs (Figure 1E). CD90, NG2, PDGFR-β were localized to the cell membrane, whereas α-SMA was intracellular, consistent with actin fiber staining.

### High glucose levels do not alter abEPC growth and migration

**Figure 2:**
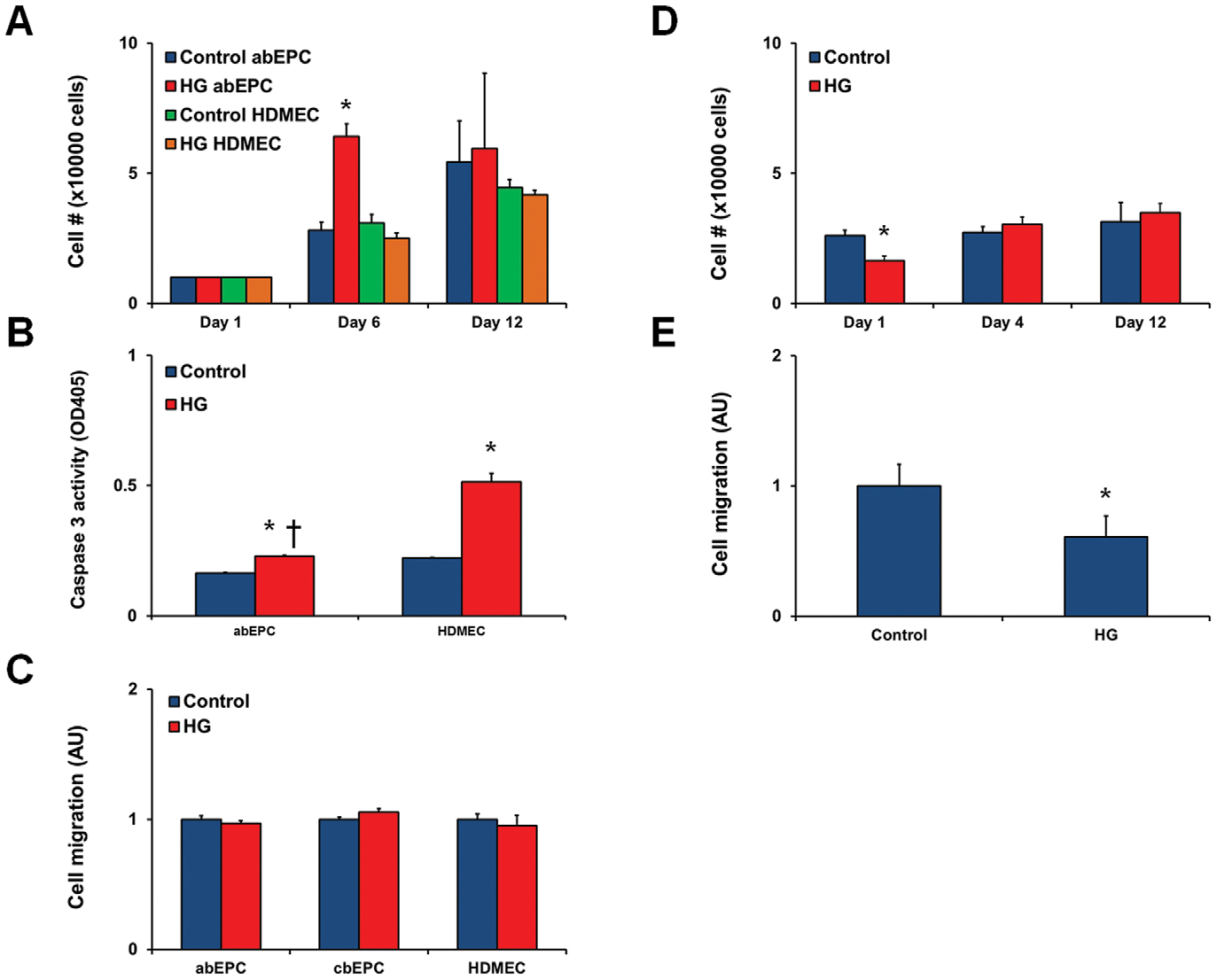
Cells were cultured in 5 mmol/L (control) or 25 mmol/L (high glucose; HG) glucose throughout the completion of each cellular activity assay. In all graphs, values indicate mean ± SEM. (A) Growth of abEPCs was assessed as cell viability over a 12 day period in high serum media (EBM2/20% FBS). A spike in activity occurred in the hyperglycemic group at day 6 (*p<0.05 compared to cells in control media). However, abEPCs showed no significant differences between control and HG-treated groups over long-term culture. (B) Caspase-3 activity level was measured in abEPCs and HDMECs exposed to control or HG media (in EBM-2/1% FBS) for 24 hours [*p<0.05 compared to respective control media; †p<0.05 compared to HDMECs in HG media; n = 3]. (C) A 24-hour migration assay was performed on abEPCs, assessed as their ability to migrate through an 8-µm pore with bFGF as the chemoattractant. HG had no effect on the migratory abilities of abEPCs, cbEPCs, and HDMECs. (D) Growth of bmMPCs was assessed as cell viability over a 12 day period in high serum media (DMEM/20% FBS). HG-bmMPCs showed significantly less growth at day 1 as compared to control (*p<0.05). However, growth over long-term culture appeared to be unaffected by HG. (E) Migration of bmMPCs was assessed in the presence of 10% FBS as the chemoattractant. Exposure of bmMPCs to HG significantly reduced the migratory ability towards FBS (*p<0.05).

We wanted to know whether acute and chronic exposure to high levels of glucose would alter abEPC growth. We cultured abEPCs for a period of 12 days in EBM-2/20% FBS supplemented with either 5 mmol/L glucose (control) or 25 mmol/L glucose (high glucose, HG). We chose to culture the cells in normal serum levels because this setting would be reminiscent of the *in vivo* conditions, and allow us to study the effect of chronically elevated glucose levels without the confounding toxicity associated with serum-free media. Our results show that the growth of abEPCs, and even HDMECs, is not affected by high levels of glucose (Figure 2A). An increase in total cell number was noted at day 6 in the abEPCs. However, this increase was normalized (relative to control media containing normal glucose levels) by day 12. We then plated a high density of cells and assessed the cell capacity for survival in depleted media (1% FBS), and how the addition of high glucose might impede this process. Addition of high glucose in 1% serum media reduced the cell number at 24 hours but had no effect at day 4 (data not shown). To confirm that the reduction in cell number was due to apoptosis, we measured caspase-3 activity in the abEPCs and HDMECs. Our results show a slight but significant increase in caspase-3 activity in abEPCs (1.4x increase as compared to control media without high glucose) (Figure 2B). Interestingly, the same conditions led to a 2.3x increase in caspase-3 activity in the mature HDMECs (Figure 2B). As the cells were plated under identical conditions (media, cell density, plate coating), these data suggest that abEPCs are resistant to glucose-induced toxicity as compared to the mature HDMECs.

Next, we tested whether high glucose causes changes in bFGF-induced migration of abEPCs. We treated abEPCs and HDMECs to normal glucose or high glucose levels in EBM-2/1%FBS. The cells were seeded on transwell inserts and the lower chamber contained 10 ng/mL bFGF. Our results show no significant alteration of these cellular processes in both EC types in the presence or absence of high levels of glucose (Figure 2C).

### HG significantly alters the growth and migration of bmMPCs

MPCs were also subjected to specific cellular activity assays in order to test their functional characteristics in a high glucose setting. Similar to the abEPC experiments, long-term growth of MPCs was measured over the course of 12 days. Hyperglycemic bmMPCs showed a significant decrease in cell number on day 1 (Figure 2D). However, with long-term culture in high glucose media the growth was seemingly unaffected, as cell number had normalized to control cells by day 4. Similarly, no significant changes were observed in serum-depleted media (data not shown). We then measured bmMPC migration using a potent mesenchymal chemoattractant, FBS. Our results show that cell migration of bmMPCs was reduced by nearly 50% in the hyperglycemic group after 24 hours (Figure 2E).

### Effect of glucose on cellular activation, matrix protein expression, and redox-sensitive enzymes

**Figure 3:**
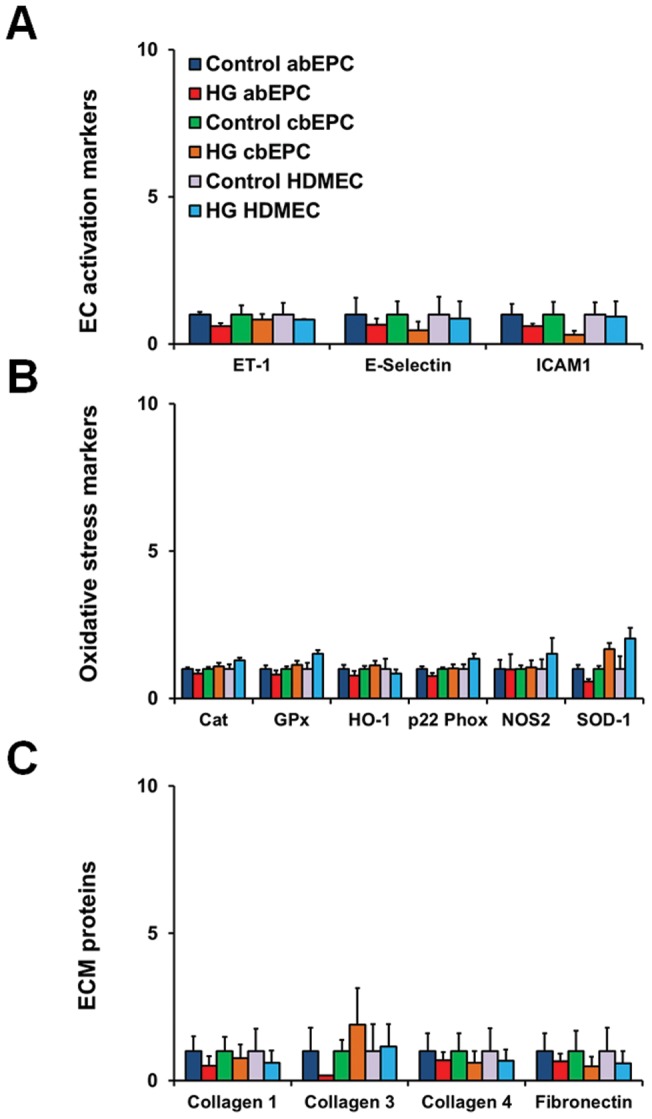
Gene expression profiles of abEPCs. Cells were cultured in 5 mmol/L (control) or 25 mmol/L (high glucose; HG) glucose for up to 14 days. RNA was isolated at day 1, day 3 (data not shown), and day 14 (data not shown) in order to assess changes in gene expression through qRT-PCR. (A) Endothelial cell activation was examined through expression of vasoconstrictor endothelin-1 (ET-1) and adhesion molecules E-Selectin and ICAM1. Contrary to what we expected, HG-treated abEPCs, cbEPCs, and HDMECs showed no significant changes in ET-1, E-selectin, and ICAM1 as compared to controls. (B) A panel of known oxidative stress markers were examined in abEPCs, cbEPCs, and HDMECs. (C) The extracellular matrix (ECM) protein profile of abEPCs, cbEPCs, and HDMECs was examined by comparing levels of fibronectin, collagens 1,3, and 4. No significant alteration in ECM proteins was noted in abEPCs, cbEPCs, and HDMECs.

**Figure 4:**
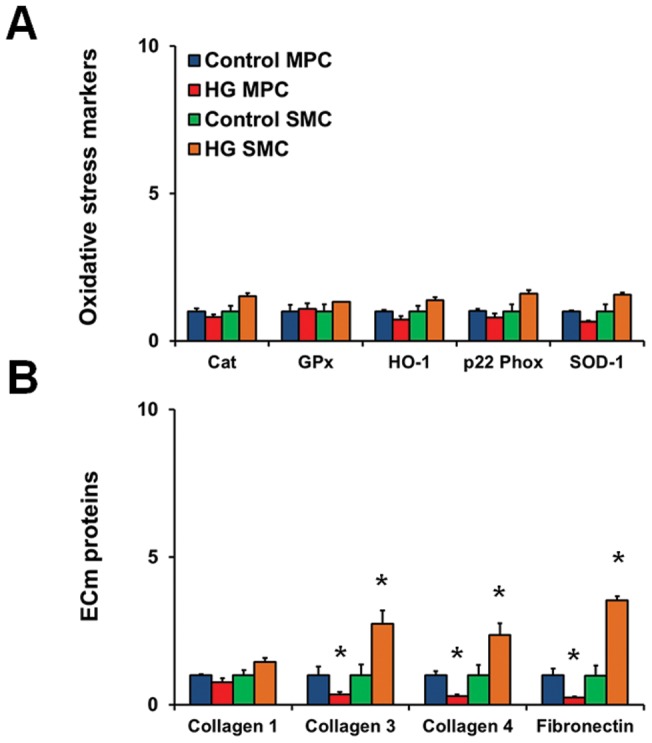
Gene expression profiles of bmMPCs. Cells were cultured in 5 mmol/L (control) or 25 mmol/L (high glucose; HG) glucose for up to 14 days. RNA was isolated at day 1, day 3 (data not shown), and day 14 (data not shown) in order to assess changes in gene expression through qRT-PCR. (A) Oxidative stress marker expression in bmMPCs and uaSMCs showed no significant changes upon exposure to high levels of glucose. (B) HG induced an increase in the production of matrix proteins in mature uaSMCs, but caused downregulation of these same markers in the bmMPCs [*p<0.05 compared to control media; n = 3].

We have previously shown altered cellular activity of ECs in high glucose conditions and in target organs of diabetic complications [24]–[26]. These altered activities include changes in the redox enzymes, expansion of extracellular matrix, and vasoactive factor alteration. We wanted to determine whether long-term culture of vascular progenitor cells would lead to similar alterations. Therefore, we cultured the cells in complete media (EBM-2/20% FBS with or without 25 mmol/L glucose) and performed a gene expression analyses. We profiled key cell activation genes (endothelin-1, ET-1; E-selectin; and intercellular adhesion molecule-1, ICAM-1), matrix protein genes (collagens 1,3,4; and fibronectin), and oxidative stress genes (catalase, Cat; glutathione peroxidase, GPx; heme oxygenase-1, HO-1; NADPH oxidase, p22 Phox; inducible nitric oxide synthase, NOS2; and superoxide dismutase-1, SOD-1). Interestingly, no significant effect of high levels of glucose was noted in endothelial cell activation, oxidative stress parameters, and matrix proteins at day 1, day 3, or day 14 (Figure 3). Analysis of bmMPCs also showed no changes in oxidative stress markers (Figure 4A). Significantly increased levels of matrix proteins (collagen 3, collagen 4, and fibronectin) in high glucose-treated uaSMCs were seen (Figure 4B). These same matrix protein mRNA levels were significantly reduced in the bmMPCs.

### Altered differentiation potential of MPCs

**Figure 5:**
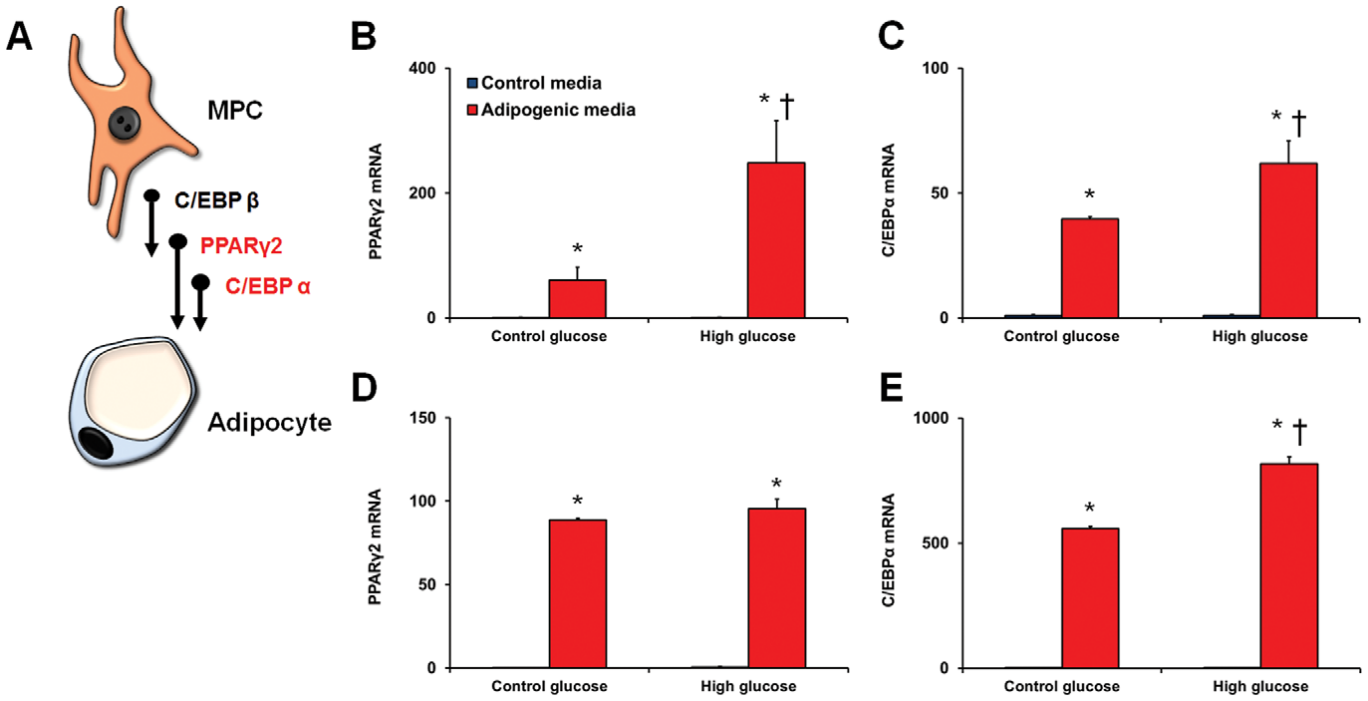
Differentiation of bmMPCs into adipocytes. bmMPCs were cultured in 5 mmol/L (control glucose) or 25 mmol/L (high glucose; HG) glucose for 7 days prior to differentiation and assessed for induction of PPARγ2 and C/EBPα (A). HG-treated bmMPCs increased adipogenesis at day 7, as demonstrated by upregulated PPARγ2 levels (B) and C/EBPα (C). Analysis of cells at day 14 showed increased PPARγ2 upon differentiation but no differences between control- and HG-treated cells. C/EBPα levels (E), on the other hand, were significantly higher in HG-treated cells at day 14 as compared to control glucose treated cells [*p<0.05 compared to control media; †p<0.05 compared to cells treated with control glucose + differentiation media].

**Figure 6:**
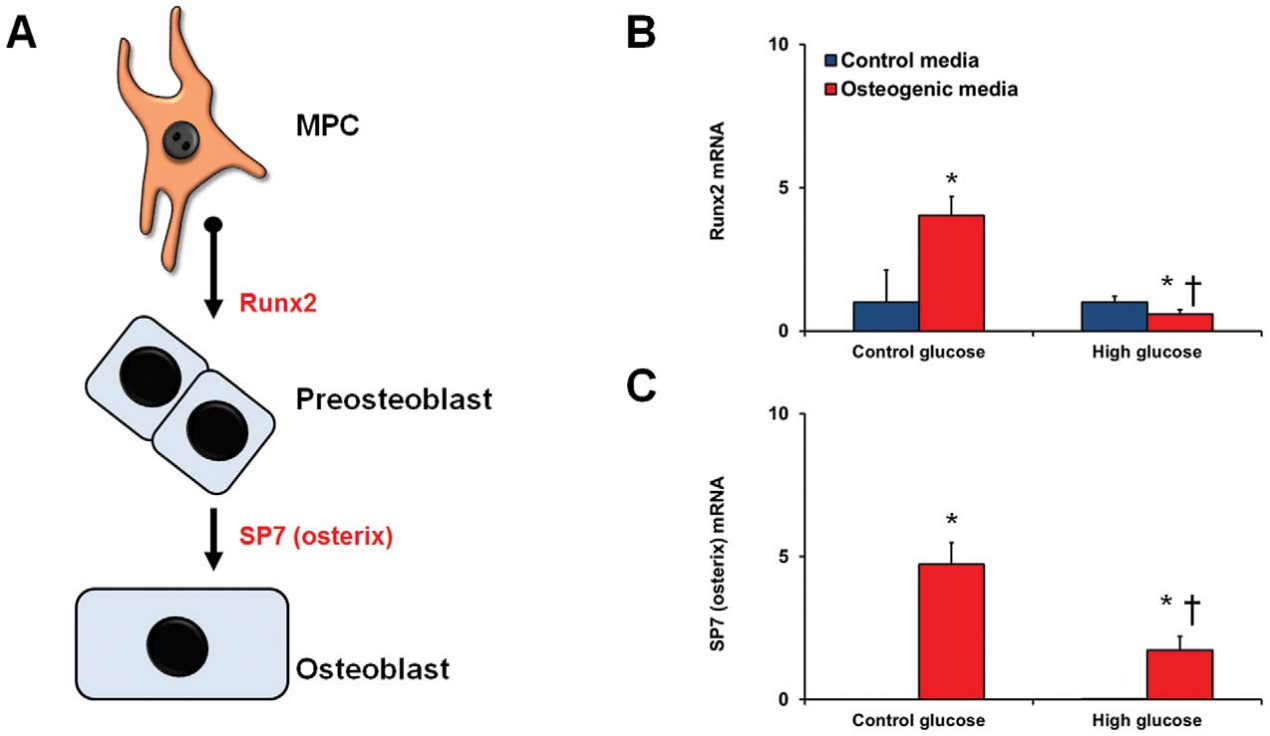
Differentiation of bmMPCs into osteocytes. bmMPCs were cultured in 5 mmol/L (control glucose) or 25 mmol/L (high glucose; HG) glucose for 7 days prior to differentiation and assessed for induction of Runx2 and SP7 (A). (B) 14-day differentiation of bmMPCs into osteocytes showed significantly depressed expression of Runx2 and SP7 in HG treated cells [*p<0.05 compared to control media; †p<0.05 compared to cells treated with control glucose + differentiation media].

**Figure 7:**
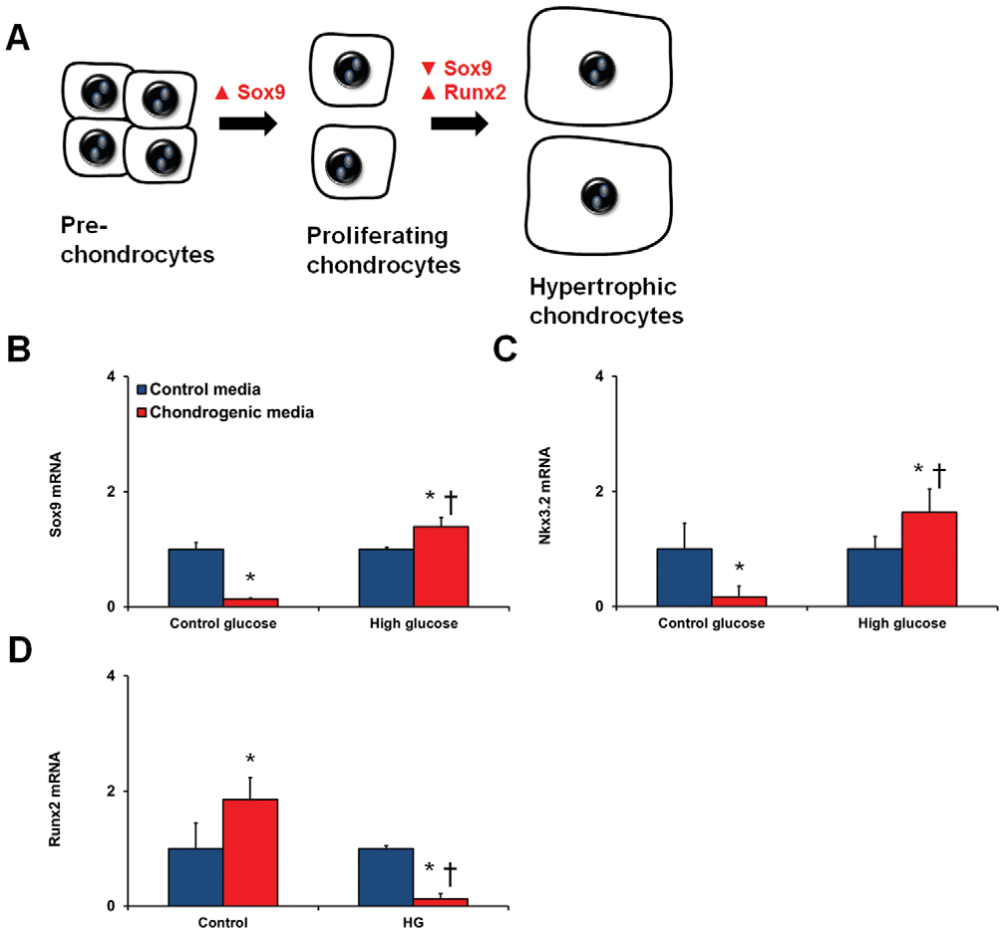
Differentiation of bmMPCs into chondrocytes. bmMPCs, pretreated with 5 mmol/L (control glucose) or 25 mmol/L (high glucose; HG) glucose for 7 days, were cultured in differentiation media and assessed for induction of chondrogenesis transcription factors (A). HG-treated bmMPCs increased expression of Sox9 (B) and Nkx3.2 (C) upon culturing in differentiation media. At this point, cells cultured in control glucose levels showed repressed levels of both Sox9 and Nkx3.2. [*p<0.05 compared to control media; †p<0.05 compared to cells treated with control glucose + differentiation media]. (D) Runx2 mRNA levels were induced after 14-day differentiation of bmMPCs. Cells treated with HG showed significantly reduced Runx2 expression [*p<0.05 compared to control media; †p<0.05 compared to cells treated with control glucose + differentiation media].

We performed mesenchymal differentiation assays on the bmMPCs to assess their ability to differentiate into adipocytes, chondocytes, and osteocytes with the addition of high glucose. The cells were pre-treated with high levels of glucose for 7 days prior to culture in the differentiation media. qRT-PCR was used to examine the expression of specific transcription factors involved in the differentiation process (adipogenesis was assessed by C/EBPα and PPARγ2 (Figure 5A); chondrogenesis by Sox9, Nkx3.2, and Runx2 (Figure 6A); and osteogenesis by Runx2 and osterix/SP7 (Figure 7A). High glucose drastically increased the differentiation of bmMPCs into adipocytes, as assessed by C/EBPα and PPARγ2 induction at day 7 (Figure 5B and 5C). At day 14, PPARγ2 levels in cells exposed to high glucose were similar to cells in the normal glucose media (Figure 5D); however, C/EBPα levels remained significantly higher (Figure 5E). Next, we assayed for osteogenic differentiation by measuring levels of osteogenic transcription factors Runx2 and osterix/SP7 (Figure 6A). Analysis of cells exposed to the differentiation media at day 14 showed that high glucose prevented Runx2 induction (Figure 6B) and significantly reduced osterix/SP7 induction (Figure 6C). Lastly, we determined whether glucose regulates chondrogenesis in bmMPCs. Sox9 mRNA levels were found to be elevated in control cells exposed to the chondrogenic media at day 7, which was not observed in cells exposed to high levels of glucose (data not shown). We then measured these transcription factors at day 14. Surprisingly, we found that cells exposed to differentiation media alone (i.e. normal glucose levels), significantly downregulated early chondrogenesis genes Sox9 and Nkx3.2 (Figure 7A–C). At this time point, cells exposed to high levels of glucose exhibited significantly higher levels of both Sox9 and Nkx3.2 (Figure 7B and 7C). Recently, it has been shown that unlike the adipogenesis-specific and osteogenesis-specific transcription factors (determined above), Sox9 plays an essential stage-specific role in chondrogenesis [23]. To test whether high glucose may be delaying these differentiation steps (Figure 7A), we measured Runx2 (late marker of chondrogenesis) in our assay. Our results show that bmMPCs induce Runx2 at day 14, which coincides with repressed Sox9 and Nkx3.2 (Figure 7D). In contrast, cells exposed to high glucose showed significantly lower Runx2 levels.

## Discussion

The present study demonstrates the differential response of vascular progenitor cell populations and mature cells to high levels of glucose. The salient findings of our study are that: 1) high levels of glucose do not alter derivation of *bona fide* EPCs and MPCs, 2) there is no significant cellular dysfunction in the abEPCs, unlike mature ECs, and 3) MPCs undergo significant cellular changes in high glucose conditions displaying altered differentiation potential.

It has been well established that high glucose causes many biochemical alterations in vascular ECs [27], resulting in impaired function [24], [27], [28]. These changes are evident when ECs are cultured in serum-free media containing high levels of glucose. Our studies do show increased caspase-3 activity (molecular correlate of apoptosis) in HDMECs exposed to high levels of glucose. Under identical conditions, caspase-3 activity level was significantly different in abEPCs as compared to HDMECs. Furthermore, this toxic effect was found to be acute (evident within 24 hours). Long-term culture of abEPCs in high levels of glucose did not alter cell activities. It should be noted that long-term assessment of cellular activity necessitated the use of serum in the media, which may mask the toxic effect of glucose. However, these conditions are consistent with early-stage changes in diabetes. Two recent studies have demonstrated a lower circulating number of EPCs (CD34+/VEGFR2+/CD31+) in both type 1 and type 2 diabetic patients [29], [30]. Further, the number of CD34+/VEGFR2+ cells has been shown to correlate with glycemic control, and negatively associate with arterial stiffness in diabetic patients [31]. This may be because of the acute effect of hyperglycemia as the EPCs are mobilized from the bone marrow. Although compelling, these recent findings also potentially take into account a reduction in hematopoietic stem/progenitor cells that share some of these same surface markers. An interesting future direction would be to pre-treat abEPCs in culture and then administer in diabetic animals to assess whether the toxic effects of hyperglycemia are evident.

Unexpectedly, we found that the progenitor population most affected by high glucose is the mesenchymal cell type. When we cultured bone marrow-derived MPCs in high glucose media, we noted a significant reduction in cell numbers at day 1. However, this effect was normalized upon continued exposure. This is a novel finding, as not much research to date has linked diabetes with changes in cells of the mesenchymal lineage. A recent study, however, has indicated that advanced glycation end products may be responsible for an increase in reactive oxygen species and subsequent decrease in proliferation and migration of bone marrow-derived MPCs [32].

MPCs exhibit remarkable plasticity, with the ability to differentiate both *in vitro* and *in vivo* into a number of mesenchymal phenotypes including those that form bone, cartilage, muscle, fat, and other connective tissues [33] [34] [35]. In addition to affecting bmMPC growth and migration, high glucose caused a very prominent change in their differentiation potential. Hyperglycemic bmMPCs exhibited enhanced adipogenesis (assessed by PPARγ2 and C/EBPα induction levels) when compared to control cells, while their ability to differentiate into alternate lineages (chondrocytes, osteocytes) was impaired. Much research to date has implicated the Wnt/beta-catenin signaling pathway as a major regulator of this process [36], [37]. Down-regulation of this pathway increases adipogenesis, whereas the use of specific Wnt proteins *in vitro* has successfully inhibited differentiation to adipocytes [38]. Whether hyperglycemia directly alters the Wnt pathway, leading to skewed differentiation into adipocytes, remains to be determined.

These studies demonstrate the differing response of mature cells and progenitor cells to high levels of glucose. We have confirmed that hyperglycemia has a toxic effect on mature ECs, inducing significant increases in apoptosis- a notion that has been well-established in diabetes research. Conversely, our studies indicate that both EPCs and MPCs may be useful therapeutic agents in diabetes. Although the cellular activity (growth and migration) of bmMPCs was disrupted initially, high glucose had little effect on both progenitor cell populations over the long-term. Therefore, increasing the number of vascular stem/progenitor cells and negating the initial toxic effect of hyperglycemia in diabetic patients may prove to be an effective means of restoring vascular homeostasis in diabetes.
